# YOLO-BP for detection of multi-scale and high intra-class variation electrode cap defects in resistance spot welding

**DOI:** 10.1371/journal.pone.0347336

**Published:** 2026-04-20

**Authors:** Xiaomin Zhao, Siow Hoo Leong, Beng Yong Lee, Sofianita Mutalib, Bangchao Qiu, Meng Lyu, Zhijun Zuo

**Affiliations:** 1 College of Automotive Engineering, Guangdong Polytechnic of Industry and Commerce, Guangzhou, China; 2 Faculty of Computer and Mathematical Sciences, Universiti Teknologi MARA, Kota Samarahan, Sarawak, Malaysia; 3 Faculty of Computer and Mathematical Sciences, Universiti Teknologi MARA, Shah Alam, Selangor, Malaysia; 4 Guangzhou MINO Equipment Co., Ltd., Guangzhou, China; 5 School of Mechanical and Automotive Engineering, South China University of Technology, Guangzhou, China; Galgotias University, INDIA

## Abstract

Inspection of electrode cap surface conditions is crucial in resistance spot welding. Current solutions largely rely on manual inspection or sensor-based approaches, with limited exploration into vision-based approaches. This paper proposes a vision-based defect detection framework, YOLO-BP, for inspecting electrode cap tip surface defects, particularly the black burn mark defects that exhibit high variability in appearance. The key contribution is the incorporation of a new attention mechanism, BiLevel Spatial Selective Attention (BSSA), which synergistically integrates hierarchical and selective attentions within the YOLOv13 architecture. The hierarchical attention utilizes BiLevel Spatial Attention Module (BSAM) to strengthen extraction of global defect geometry when defects are spatially spread, and local defect irregularities when defects are small or dispersed. Meanwhile, the selective attention employs Partial Convolution (PConv) module to selectively detect defect-sensitive regions. To facilitate evaluation of defect detection, a new annotated Electrode Cap Tip Surface Defect (ECTSD) Dataset containing diverse electrode cap tip surface defects is constructed. The results show that YOLO-BP, achieves 66.5% mAP@0.5 and 31.3% mAP@0.5-0.95, corresponding to improvement of 2.9% and 2.1% respectively over the YOLOv13 baseline, along with gains of 1.7% in recall and 1.1% in F1-Score are observed, while maintains efficient inference time of 0.8 ms. Compared with YOLOv13 integrated with the Convolutional Block Attention Module (CBAM), YOLO-BP demonstrates a clear advantage in detecting the diverse defects, achieving 4.5% higher in mAP@0.5 and 8% higher in mAP@0.5-0.95. Furthermore, it attains the highest F1-Score of 67.7% when benchmarked against YOLOv5 through YOLOv12. The superiority of entire defect detection indicating the effectiveness of YOLO-BP in minimizing false negatives, underscoring its practical application in industrial inspection for defects.

## Introduction

Resistance spot welding (RSW) is used to join sheet metal by utilizing a combination of pressure and heat generated by an electric current to the weld area. This technique forms a weld nugget where the electrodes contact the metal surfaces. It is extensively employed in industries such as automotive and aviation manufacturing, particularly for bonding thin metal sheets [1–3]. Inspection of weld quality in RSW is critical to ensure structural integrity and performance. Non-destructive testing methods are widely employed to evaluate weld quality without compromising the component, for instance, ultrasonic testing [4], radiographic testing [5], eddy current testing [6], thermographic inspection [7]. Data driven modelling for weld quality and defect prediction in RSW [8,9], as well as simulation for optimized process parameters of welding [10] emerges in recent years. These non-destructive testing methods offer advantages such as real-time evaluation, high accuracy, and the ability to inspect large volumes of welds efficiently. By integrating these non-destructive methods, manufacturers can ensure consistent weld quality, reduce production costs, and enhance the reliability of welded components, ultimately contributing to safer and more durable products.

The weld quality in RSW is highly dependent on the conditions of the electrode cap used during the spot-welding process [11]. The surface state of the electrode cap directly influences weld penetration, bead morphology, and overall joint integrity. A primary challenge is the degradation of the electrode cap through wear and contamination at the tip and side surfaces after welding, including oxides and stamping oil residues at the melted areas on the tip [12], black deposit resulting from burned contaminants at the contact surface [13]. These imperfections imprint left on the electrode cap surface deteriorate the quality of subsequent welds [14,15]. Consequently, a rigorous and regular maintenance regimen of electrode cap is critical for ensuring weld quality and consistency, which includes grinding or dressing, polishing, cleaning, and followed by quality inspection of the surface conditions. An automated system utilizing digital fibre-optic sensor has been introduced by analyzing the reflected light from the electrode cap surface to assess its condition [16]. However, research into alternative methods for inspecting the condition of electrode cap surface remains limited.

This paper proposes to use computer vision-based approach for inspecting surface condition of electrode cap, or in other words, detecting defects of electrode cap. To the best of the authors’ knowledge, no prior studies have addressed the problem using similar approaches in the existing literature. It is motivated by the rapid advancement of deep learning and computer vision technologies, which offer promising alternatives for automated defect detection, and revolutionize industrial approaches for quality control and inspection [17]. Recent studies have demonstrated that integrating the YOLO (You Only Look Once) algorithm into manufacturing quality control systems can achieve high precision and recall in anomaly detection [18,19]. Ongoing enhancements to the YOLO architecture have led to successive improvement in surface defects across various manufacturing domains. For example, an improved version of YOLOv5 has been applied to detect surface defects in printed circuit boards [20]. Enhanced YOLOv8 models have been utilized for steel surface inspection during manufacturing processes [21,22], as well as for defect detection on metal surfaces [23] and metal cylinder joints [24]. Further, YOLOv9 has been adapted for monitoring surface quality in metal-based directed energy deposition processes [25], while YOLOv10 has been improved for photovoltaic manufacturing applications [26]. Subsequent versions, such as YOLOv11 and YOLOv12, have also been enhanced to address surface defect detection in steel surfaces [27] and printed circuit boards [28], respectively. Despite these promising developments, the adaptability of deep learning-based solutions in industrial applications continues facing challenges, particularly in adapting to complex industrial environments and previously unseen defect types.

**Fig 1:**
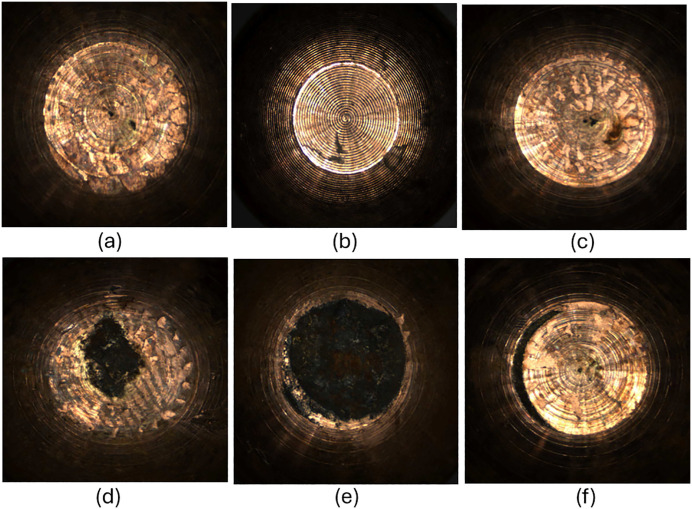
Defects of electrode cap tip surface in multi-scale and high intra-class variability. Black burn marks that are: (a) tiny dots, (b) elongated stripe, (c) scattered, (d) partial patch, (e) full patch, (f) curved stripe at edge.

This paper focuses on the black burn mark defect of electrode cap tips in RSW. It is a common external defect resulting from a combination of interactions between excessive heat, insufficient electrode force and cooling, surface contaminations, electrode tip wear and alloying [29,30]. The burn mark is a visible buildup that appears black on the electrode cap tip surface. The main challenge of using computer vision-based approach to detect the black burn mark is the variability in its appearance. A black burn mark defect could be inconsistently distributed on the electrode cap tip: in the center, at the edge, or scattered all over the tip. Also, it could appear in patches, tiny dots, elongated stripes, or curved stripes. In other words, each burn mark has a different visual signature as shown in Fig 1. It leads the detection of burn mark defects to a challenge involving detection of objects with multi-scale and high intra-class variation in terms of spatial distribution.

Accurate detection of multi-scale objects remains a significant challenge in automated defect detection. To address this, recent advancements have leveraged attention mechanisms to enhance the representational capacity of convolutional neural networks (CNNs). These mechanisms improve model performance by emphasizing salient features and suppressing background noise, making them effective for detecting objects of varying sizes and complexity [31,32]. A notable example is the Multi-Scale Spatial Pyramid Attention (MSPA) module, which extracts spatial information at multiple scales through an adaptive fusion mechanism that integrates structural regularization. Its modular design allows for easy integration into various CNN backbones [32]. Similarly, YOLO-based models with enhanced attention and feature fusion components have shown strong performance in detecting multi-class and multi-scale surface defects [33,34]. In aluminium defect detection, AMMENet incorporates a Parallel Residual Attention Module (PRAM) to suppress background interference, alongside a Multilevel Semantic Fusion Module (C2f-MFF) to integrate multi-scale features [35]. For steel surface defect detection, EAD-YOLOv10 uses C2f_EMSCP, combining multi-scale convolutions with position-aware attention to improve sensitivity [36], MPA-YOLO introduces a Multi-Path Convolution Attention Module and an auxiliary detection head for enhanced feature extraction and background separation [22], FMV-YOLO employs a Multi-Scale Attention Fusion (MSAF) module and NWD loss to enhance localization accuracy of small-scale defects [27]. In metal surface defect detection, IAMF-YOLO, an Information Augmentation Network and a Parallel Multi-Scale Feature Pyramid Network (PMFPN) address downsampling losses and improve detection of small defects [24], SLF-YOLO includes a SC_C2f module with channel gating and a Light-SSF_Neck for improved multi-scale feature fusion and morphological extraction [23].

On the other hand, the challenge of high intra-class variation has been mitigated by training a flow model on the extracted high-level features which contain semantic information extracted from image datasets [37]. The flow model is extended by using densities estimated from normalizing flows [38]. To tackle significant visual difference exists in same class of skin lesions in dermoscopic images, a feature updating mechanism is used to capture global features guided intra-class feature concentrating [39]. A balance augmentation method for achieving a balanced number of annotation boxes in YOLO is used to improve detection of apples with high intra-class variation [40]. However, improvement on YOLO network for detecting high intra-class variation objects is scarce.

To address the challenges posed by multi-scale and shape variation in spatial distribution, as well as representation of small object features in electrode cap tip surface defect detection, this paper proposes YOLO-BP, a vison-based defect detection built upon YOLOv13 [41] and integrated with a newly designed BiLevel Spatial Selective Attention (BSSA). The BSSA jointly incorporates hierarchical attention and selective attention during feature extraction and prediction stages respectively, distinguishing it from prior studies. The hierarchical attention utilizes BiLevel Spatial Attention Module (BSAM) at the backbone to enhance coarse-grained selection followed by fine-grained attention through Bi-Level Routing Attention (BRA). It emphasizes global defect geometry when defects are spatially spread, and local defect irregularities when defects are small or dispersed. Meanwhile, the selective attention employs Partial Convolution (PConv) module to selectively highlight defect-sensitive regions while suppressing noises at the detection head. The main contributions of this paper can be summarized as follows:

[1] A new annotated Electrode Cap Tip Surface Defect Dataset (ECTSD Dataset) with diverse appearance of black burn mark defects is constructed, providing a benchmark for electrode cap tip quality inspection in resistance spot welding and relevant industrial defect detection research.
[2] A novel BiLevel Spatial Selective Attention (BSSA) is proposed, which jointly integrates hierarchical attention and selective attention at different stages of the detection pipeline to enhance feature extraction and representation, as well as prediction.
[3] A vision-based defect inspection framework, YOLO-BP, is developed and applied to electrode cap tip surface inspection, extending the applicability of object detection models to this domain.
[4] Experiments demonstrate that the proposed YOLO-BP exhibits superior performance when detecting intra-class defects that appear in high variability while maintaining real-time efficiency.

### Acquisition of electrode cap tip surface defect (ECTSD Dataset)

The electrode cap samples were collected from an intelligent manufacturing factory in China. During the welding process of manufacturing, it was noticed that burn marks appear on tip surface of the electrode caps. To ensure welding quality, these burn marks were removed through grinding equipment. However, at certain times, the burn marks were not completely removed. The electrode cap samples with such defects from the production line were collected for this study.

The original top-down view electrode cap images were captured using a HIKVISION MV-CE003–20GM industrial camera mounted securely on a C-stand and connected to a computer with software setting for clear imaging. The electrode cap was positioned directly below the camera in the center of the frame to capture the tip region of the electrode cap, resulting in a total of 260 tip surface images, all with a resolution of 1280 × 1280.

**Fig 2:**
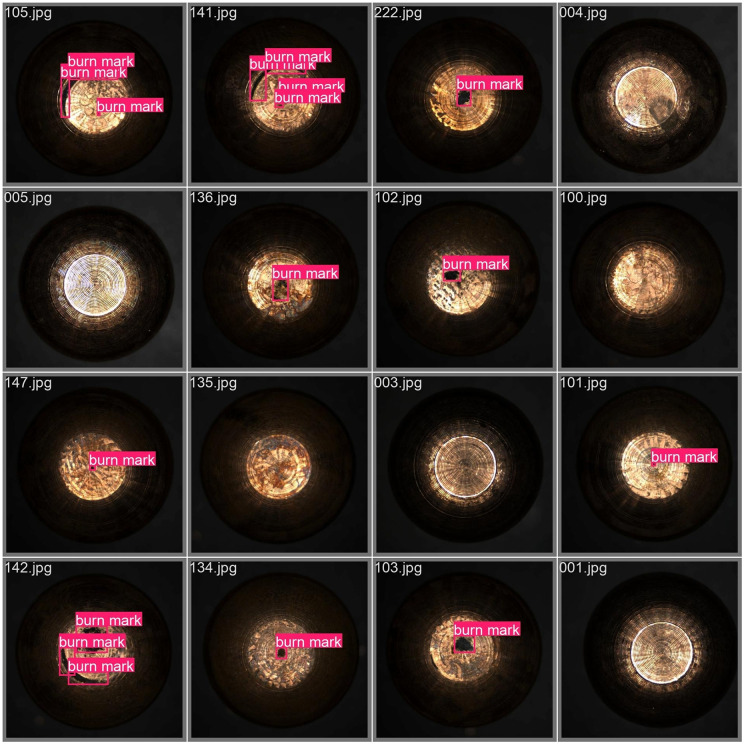
Representative samples of burn marks in the ECTSD dataset show differences in sizes and shapes.

The burn mark defects of the images were annotated using the LabelImg software with label “burn mark”, and the annotation files were saved in YOLO format. This image dataset is referred to Electrode Cap Tip Surface Defect Dataset (ECTSD Dataset). A uniform random sampling strategy was adopted to split the images into training, validation, and testing sets with a ratio of 7:2:1, which is widely considered in surface defect detection [42–44]. Representative images of the electrode cap with labels of burn mark are shown in Fig 2. The burn mark defects are varied in size and spatial distribution, and special interest is on the burn marks in stripe form at the edge of the electrode cap tips.

## Proposed YOLO-BP

The proposed YOLO-BP adopts YOLOv13 [41] as the baseline network, which was the latest version of YOLO at the time the research was conducted. The architecture of YOLOv13 is enhanced for multi-scale effectiveness and improved detection of small objects, making it highly relevant for identifying electrode cap defects of this study. YOLO-BP is incorporated with a novel attention mechanism, BiLevel Spatial Selective Attention (BSSA), to enhance the detection of multi-scale and high intra-class variation electrode cap tip surface defects. The architecture of YOLO-BP is described in the following sub-sections.

### The baseline network YOLOv13

YOLOv13 maintains the single-stage detection framework while introducing systematic improvements to the backbone, feature fusion structure, and detection head. In the backbone, the A2C2f module is integrated to enhance feature extraction capacity and training stability. The neck adopts an improved Feature Pyramid Network (FPN) combined with a Path Aggregation Network (PAN), enabling efficient fusion of high-level semantic information and low-level spatial details. For the detection head, YOLOv13 employs a decoupled design that separates classification and regression branches, coupled with an optimized regression loss function to further improve localization accuracy.

Two key innovative modules, Hyper-Aggregated Cross-scale Enhancement (HyperACE) and Full-Precision Anchor-free Decoupling (FullPAD), are introduced in YOLOv13. HyperACE strengthens cross-scale feature interactions through efficient multi-level aggregation, allowing the network to achieve stronger scale adaptability while remaining lightweight, thereby improving the detection of small and densely distributed objects. FullPAD introduces a full-precision anchor-free decoupling strategy in the detection head, where target centers and bounding boxes are directly predicted. This eliminates the redundant computation and biases associated with anchor matching, while the full-precision constraint ensures higher regression accuracy.

**Fig 3:**
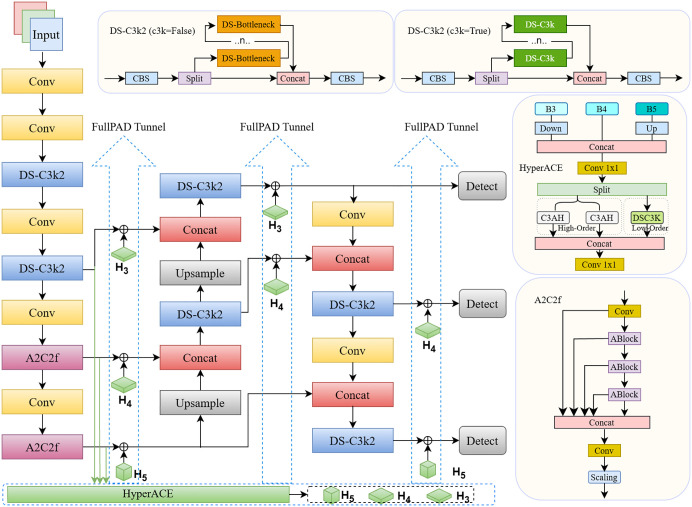
Network architecture of YOLOv13.

Experimental results on the COCO dataset, Pascal VOC, and industrial scenario datasets demonstrate that YOLOv13 achieves superior accuracy and recall compared with other earlier versions of YOLO, while exhibiting stronger robustness under complex background conditions [41]. The overall architecture of YOLOv13 is illustrated in Fig 3.

### BiLevel spatial selective attention (BSSA)

A new attention mechanism BiLevel Spatial Selective Attention (BSSA) is proposed to jointly leverage hierarchical and selective attentions, enabling more effective modelling of the diverse multi-scale defect characteristics. It consists of two modules, the BiLevel Spatial Attention Module (BSAM) for hierarchical attention, and the Partial Convolution (PConv) module for selective attention.

#### Hierarchical attention.

**Fig 4:**
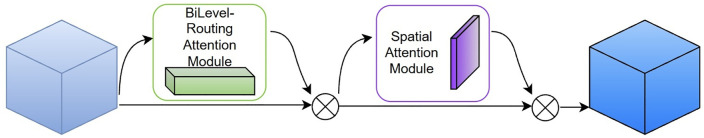
BSAM block.

The convolutional feature extraction process in YOLO treats all regions equally, lacking adaptive focus on critical information. In the Electrode Cap Tip Surface Defect Dataset (ECTSD Dataset), the burn mark defect targets vary significantly in size and spatial distribution. As a result, convolutional layers may disperse limited computational resources to irrelevant regions, making small or edge-located defects difficult to detect accurately. Attention mechanisms can adaptively assign weights across channel and spatial dimensions, emphasizing target-related features while suppressing redundant background information, thereby enhancing the discriminative power of feature representation. Inspired by the multi-scale object detection capability of the BiLevel Spatial Attention Module (BSAM) [45], which replaces the channel attention mechanism in Convolutional Block Attention Module (CBAM) [46] by Bi-Level Routing Attention (BRA) [47], as shown in Fig 4, this study adopts the BSAM module for feature representation of electrode cap tip defects.

**Fig 5:**
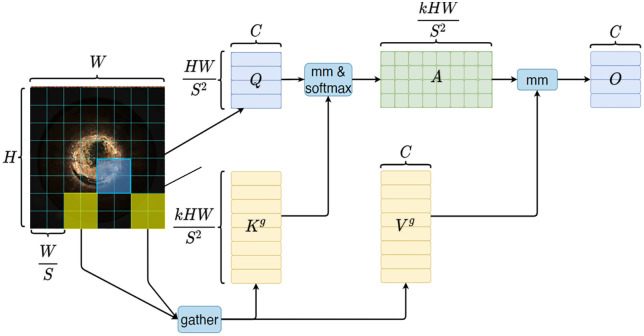
BiLevel routing attention module.

The main advantage of the BSAM is that its channel attention is enhanced using fine-grained (intra-group) and coarse-grained (inter-group) attention routing of BRA, thus strengthens the representation of what the object is. The BRA introduces a top-down hierarchical mechanism that performs coarse-grained selection at the region level, followed by fine-grained attention computation at the token level as illustrated in Fig 5. At region level, the routing process discerns larger-scale structural irregularities and contextual dependencies that distinguish a defect shape from another. It mitigates the ambiguity of the same defect type that may vary in shape, size and orientation. At the token level, it distinctively attends to detail-rich features where subtle textural or geometric deviations often manifest.

During the computation process, given an input feature map $\begin{array}{c} X\in R^{H\times W\times C} \end{array}$, it is first partitioned into S × S non-overlapping regions, with each region containing $\frac{\text{HW}}{{\text{S}}^{\text{2}}}$ feature vectors. Subsequently, linear projections are applied to obtain the query (*Q*), key (*K*), and value (*V*) representations. Unlike conventional attention mechanisms that compute similarities across all tokens, BRA first applies average pooling within each region to derive the region-level representations. The affinity between regions is then computed as shown in Eq. (1):


$$A_{r}=Q_{r}\overset{T}{\underset{r}{K}}$$ (1)

where *A*_*r*_ and *K*_*r*_ denote the region-level query and key matrices, respectively.

After obtaining the region-level affinity matrix *A*_*r*_, BRA performs Top-*k* routing to retain only the most semantically relevant regions for each query region. Formally, for each region *i*, the routing index set *I*_*i*_ is defined as Eq. (2):


$$\begin{array}{c} I_{i}=\text{Top}-k\left(A_{r,i}\right), \end{array}$$ (2)

where *A*_*r,i*_ denotes the affinity scores between region *i* and all other regions, and Top-*k* selects the indices of the top-*k* highest scoring regions.

Once the routing indices are determined, the corresponding key and value tokens from the selected regions are gathered to form the reduced candidate sets *K*_*g*_ and *V*_*g*_. The token-level attention is then performed as Eq. (3):


$$\begin{array}{c} O=\text{Softmax}\left(\frac{Q\overset{T}{\underset{g}{K}}}{\sqrt{d}}\right)V_{g}, \end{array}$$ (3)

where *d* is the dimension of the query/key embeddings.

To further enhance local spatial information, a Local Context Enhancement (LCE) term based on depth-wise convolution is added, and the final output is expressed as Eq. (4):


$$O^{\prime }=O+\text{LCE}(V).$$ (4)

This hierarchical design enables BRA to dynamically route queries to the most relevant regions, learning to prioritize the most informative features across scales, enabling robust feature extraction even the defects exhibit significant variability while retaining both long-range dependencies and local structural details. BRA constructs a sparse region-level routing index matrix where each region only interacts with the most relevant regions rather than performing computations with all regions globally. This design achieves dynamic and query-aware sparsity while preserving the ability to model global dependencies. The hierarchical bi-level attention emphasizes global defect geometry when defects are spatially spread, and local defect irregularities when defects are small or dispersed, ultimately improving the capacity to capture defect features under intra-class diversity.

The spatial attention branch of the BSAM module as illustrated in Fig 4, functions as refinement component that complements the BRA mechanism. It operates along the spatial dimensions of the feature map, strengthening the spatial positioning of salient features and providing distribution of informative regions. For the intermediate feature maps $\begin{array}{c} F\text{'}\in R^{H\times W\times C} \end{array}$ refined by the BRA module, cross-channel max pooling and cross-channel average pooling are applied, as defined in Eq. (5) and Eq. (6) respectively. These two types of pooled representations capture complementary spatial information: the max pooling emphasizes the most salient features, while the average pooling retains the overall contextual structure of the spatial distribution.


$$\begin{array}{c} M_{max}=MaxPool(F)\in R^{H\times W\times \text{1}} \end{array}$$ (5)

$$\begin{array}{c} M_{avg}=AvgPool(F^{\prime })\in R^{H\times W\times \text{1}} \end{array}$$ (6)

Subsequently, the two maps are concatenated and processed by a standard convolution layer to produce a spatial attention mask, as shown in Eq. (7).


$$\begin{array}{c} A_{S}=\sigma \left(Conv_{\text{7}\times \text{7}}\left(Concat\left(M_{max}{,M}_{avg}\right)\right)\right) \end{array}$$ (7)

where $\begin{array}{c} Conv_{7\times 7} \end{array}$ denotes a convolution with a 7x7 kernel, and *σ* is the sigmoid activation function to normalize the attention weights between 0 and 1. Finally, the spatial attention mask $\begin{array}{c} A_{S} \end{array}$ is applied to the BRA refined feature map $\begin{array}{c} F\text{'} \end{array}$ via element-wise multiplication, as shown in Eq. (8).


$$\begin{array}{c} F_{out}=F\text{'}⊗A_{S} \end{array}$$ (8)

In the BSAM module, while the BRA mechanism robustly captures salient features, the spatial attention component explicitly encodes the spatial distribution of the salient features, thus, enforcing precise feature location. These dual enhancement of the feature representations, thereby facilitating comprehensive feature optimization for the multi-scale and high intra-class variation defects of in study.

**Fig 6:**
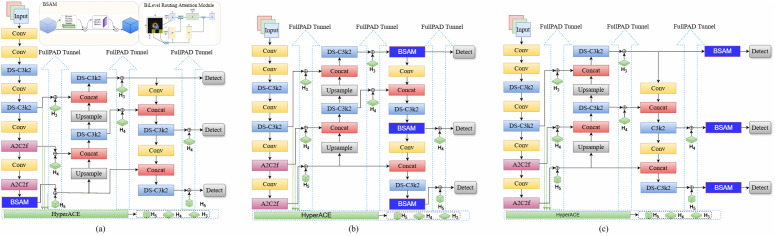
YOLOv13-BSAM network. Integration of the BSAM module into the: (a) backbone of YOLOv13, (b) neck of YOLOv13, (c) head of YOLOv13.

Attention modules are commonly integrated into the backbone or neck of YOLO for feature extraction or feature fusion respectively and show significant improvement in object detection. For instance, embedded in the backbone [24,27], neck [23,48], backbone and neck [22,36]. Research on embedding attention modules in YOLO has been extended to the head for refinement of final prediction features [49,50]. This study incorporates the BSAM module into different positions of the network of YOLOv13 to examine its role across various feature hierarchies as illustrated in Fig 6. It aims to determine the optimal position of BSAM to be embedded in the proposed YOLO-BP.

#### Selective attention.

While BSAM enriches semantic representation and discrimination, Partial Convolution (PConv) module [51] is proposed as a substitute of the conventional full-channel convolution in the detection head to improve spatial precision by selectively updating only salient regions during convolution. The integration of PConv also aims to suppress noise propagation from irrelevant texture and highlights defect-sensitive regions which is critical when defects exhibit high intra-class variation. PConv and its variants have shown promising results in reducing computational demands [52,53]. While its effects have been investigated within backbone modules [54,55], this paper explores its potential application in head modules to enhance attention mechanism at prediction of electrode cape tip defects.

**Fig 7:**
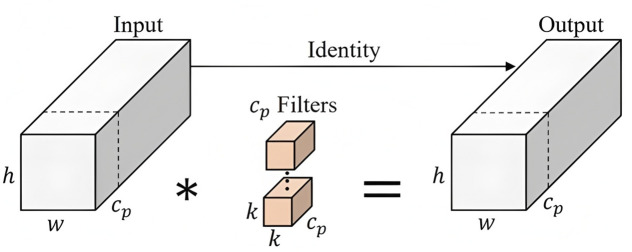
PConv block.

The working principle of PConv is illustrated in Fig 7. It applies a standard convolution only to a subset of the input channels for spatial feature extraction, while the remaining channels are left untouched and directly propagated. Specifically, only a fraction filter *c*_*p*_ of the total channels 𝑐 is used for the convolution process, where the typical partial ratio $\begin{array}{c} =\frac{c_{p}}{c}=\frac{\text{1}}{\text{4}} \end{array}$. The remaining channels, *c* – *c*_*p*_, are retained in their original form and passed through to the next layer without convolution. This approach ensures that the unused channels, although not directly involved in the spatial feature extraction, remain intact to preserve valuable information for subsequent layers. Integrating PConv into the detection head of model does not trade-off detection accuracy for efficient computation. Small objects often rely heavily on the spatial details provided by high-resolution feature layers, yet these layers are computationally expensive. PConv addresses this by applying convolution only to a subset of channels in high-resolution layers, thereby preserving the spatial details required for small object detection while avoiding the high cost of full-channel convolution. This mechanism not only improves detection efficiency but also maintains high accuracy for small objects.

#### Overall architecture of YOLO-BP model.

**Fig 8:**
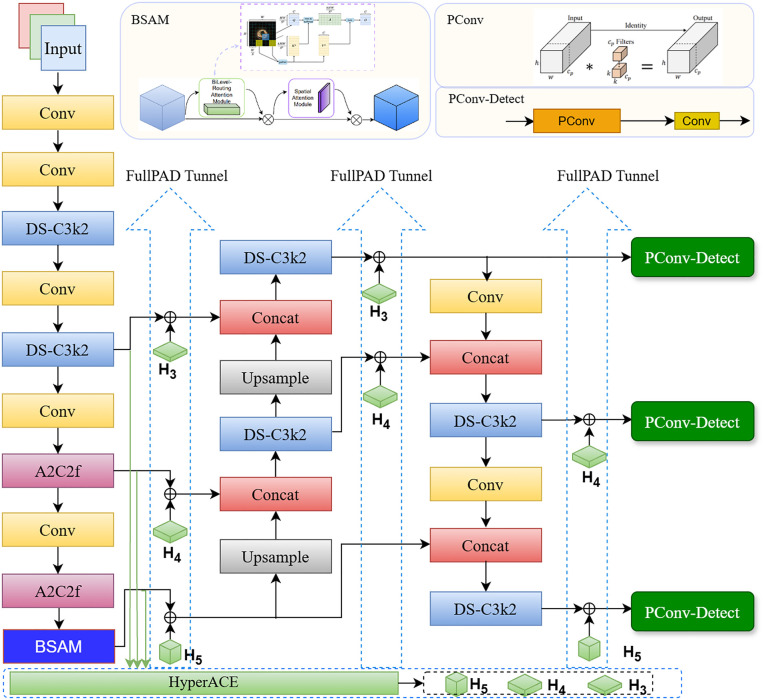
The overall network architecture of YOLO-BP.

Integrating the BSSA into YOLOv13, this paper proposes the YOLO-BP algorithm as illustrated in Fig 8 to address the challenges of defects detection in electrode cap tip surface. The combined architecture leverages hierarchical attention for semantic robustness and discrimination across multi-scale and high diversity defect appearances, while selective attention emphasizes spatial discrimination within defect-sensitive refinement and noise suppression. Based on the results on the optimal position of BSAM in YOLOv13 in the Subsection of Optimal position of BSAM in YOLOv13 (Results and Discussion Section), BSAM is embedded into the backbone of YOLO-BP to reinforce the representation of salient regions at an early stage of feature extraction, and improve the network’s ability to capture critical semantic information while effectively suppressing redundant background interference. While maintaining global dependency modeling, BSAM enhances the discriminative quality of the backbone features, thereby providing more informative inputs for subsequent multi-scale feature fusion. In the detection head, conventional full-channel convolution is replaced by PConv. The PConv applies convolution only to a subset of channels while directly propagating the remaining channels, thus reducing computational redundancy and memory overhead without compromising the extraction of essential spatial features for prediction. This design enables the detection head to perform classification and bounding box regression in a more lightweight manner, while preserving sensitivity to small objects and high-resolution features and strengthening feature localization by enforcing spatial sparsity.

## Experiment

### Experimental environment and parameters

The experiments were conducted on a workstation equipped with an Intel Core i9 CPU (64 GB RAM), an NVIDIA GeForce RTX 4090 GPU (24 GB VRAM), CUDA 11.3, and PyTorch 1.10.1. Multiple data augmentation strategies are jointly employed to mitigate overfitting in deep learning caused by data scarcity, including filtering operations, geometric transformations, image mixing, color space transformations, and mosaic augmentation [56,57]. Specifically, Gaussian blur is used to simulate global blur conditions, while high-contrast horizontal and vertical edge filters are applied to enhance edge feature representation. For geometric transformations, image scaling and cropping are used to adjust spatial resolution, while rotations within 0°−360°, translations along the X and Y axes, and shear transformations are applied to increase diversity in object pose and spatial distribution; horizontal and vertical flipping are further introduced to enhance directional invariance. Image mixing methods generate new samples by combining multiple images, and color space transformations enrich visual representations by adjusting brightness, contrast, and color distribution. In addition, mosaic augmentation randomly selects multiple training images and stitches them into a composite image according to a predefined spatial layout, thereby increasing diversity in object scale and spatial arrangement. Through the systematic integration of these augmentation methods, the diversity of training samples is effectively improved, the model’s reliance on local sample-specific features is reduced, overfitting during training is significantly suppressed. The models were trained using Stochastic Gradient Descent (SGD). The training parameters were set as follows: the number of epochs was 400, the input image size was 640 × 640, the batch size was 16, the initial learning rate was 0.01, the weight decay was 0.0005, and the momentum was 0.937. Throughout training, no pre-trained weights were used for any of the networks.

### Evaluation metrics

The performance of the proposed method was evaluated using common metrics in object detection tasks, including Precision, Recall, mAP@0.5, mAP@0.5–0.95, F1-Score, and FPS [58]. Precision describes the proportion of correctly detected defects among all predicted defects. Recall measures the proportion of correctly detected defects among all defects that should have been detected. AP is defined as the area under the Precision–Recall (P–R) curve, representing the prediction performance for a single class, while mAP is the mean of AP values across all classes, reflecting the overall performance of the model. F1-Score is a comprehensive metric in object detection that represents the weighted harmonic mean of Precision and Recall, effectively balancing the accuracy of positive predictions and the ability to identify actual positive samples. FPS reflects the processing speed of the model during inference. The mathematical definitions of these metrics are provided in Eqs. (9–14).


$$\begin{array}{c} Precision=\frac{TP}{TP+FP} \end{array}$$ (9)

$$\begin{array}{c} Recall=\frac{TP}{TP+FN} \end{array}$$ (10)

$$\begin{array}{c} AP=\int_{\text{0}}^{\text{1}}p(r)dr \end{array}$$ (11)

$$\begin{array}{c} mAP=\frac{\text{1}}{C}\sum_{i=\text{1}}^{c}AP_{i} \end{array}$$ (12)

$$\begin{array}{c} F\text{1}-Score=\frac{\text{2}*(Precision*Recall)}{\left(Precision+Recall\right)} \end{array}$$ (13)

$$\begin{array}{c} FPS=\frac{N}{t_{N}} \end{array}$$ (14)

Here, TP (True Positives) refers to the number of positive samples that are correctly detected, FP (False Positives) denotes the number of negative samples that are incorrectly predicted as positives, and FN (False Negatives) represents the number of positive samples that are missed by the detector. mAP@0.5 indicates the mean Average Precision (AP) across multiple classes at an IoU threshold of 0.5, which corresponds to the area under the P-R curve when IoU = 0.5, mAP@0.5-0.95 averages the AP values across multiple IoU thresholds ranging from 0.5 to 0.95 with a step size of 0.05, providing a more comprehensive and reliable evaluation of model performance under different overlap criteria, *N* is the quantity of images, *t*_*N*_ is the total time spent by the model to detect images.

## Results and discussion

### Optimal position of BSAM in YOLOv13

The BSAM module was inserted into the backbone, neck, and head of YOLOv13, respectively, and the performance varied across different positions as shown in Table 1. When BSAM was integrated into the backbone, the model achieved the best-balanced detection quality with F1-Score of 0.675, while maintaining the highest inference speed (1667 FPS). This indicates that introducing attention at the feature extraction stage can effectively enhance feature representation and improve detection performance with negligible computational overhead. When BSAM was applied to the neck, the model attained the highest Precision (0.858) among the three configurations, but Recall dropped to 0.52. Although mAP@0.5-0.95 slightly improved to 0.301, the overall mAP@0.5 (0.636) was lower than that achieved in the backbone case. Moreover, inference speed decreased substantially to 714 FPS, suggesting that inserting attention during multi-scale feature fusion may enhance local accuracy, but at the cost of reduced recall and significantly higher computational burden. When BSAM was placed in the head, Recall increased to 0.59, but Precision dropped sharply to 0.655, resulting in the lowest overall mAP@0.5 (0.619) and mAP@0.5-0.95 (0.288). The inference speed remained moderate at 909 FPS. This suggests that applying attention in the detection head may interfere with the decoupling of classification and regression branches, thereby reducing overall detection accuracy. The experimental analysis demonstrates that incorporating BSAM into the backbone yields the most favorable trade-off between accuracy and efficiency. The results are supported by [24] and [27]. However, [59] infer that incorporating too many attention modules in the backbone can introduce unnecessary complexity, potentially compromising the model’s speed and accuracy advantages. While inserting BSAM into the neck or head offers partial improvements in specific metrics, their overall performance is inferior compared to the backbone configuration. This may explain the emerging trend in some models to embed attention modules in both the backbone and the neck [22,36].

**Table 1:** Performance comparison of BSAM module inserted at three positions: Backbone, Neck, and Head.

Position	Precision	Recall	mAP@0.5	mAP@0.5-0.95	F1-Score	FPS
**Backbone**	0.828	0.57	**0.648**	0.299	**0.675**	**1667**
**Neck**	**0.858**	0.52	0.636	**0.301**	0.647	714
**Head**	0.655	**0.59**	0.619	0.288	0.621	909

### Ablation experiments

To validate the effectiveness of the proposed improvements in YOLO-BP for detection of electrode cap tip surface defects, ablation experiments were conducted using YOLOv13 as the baseline on the self-constructed ECTSD Dataset. All experimental settings were kept consistent, and no pre-trained weights were used to ensure fairness. The results of the ablation study are presented in Table 2. YOLO-BSAM denotes the model with the BSAM module integrated into the backbone of YOLOv13, YOLO-PConvHead refers to the model with the PConv module introduced into the detection head, and YOLO-BP represents the proposed detection model that integrated with BSSA.

**Table 2:** Results of ablation experiments.

Models	Precision	Recall	mAP@0.5	mAP@0.5-0.95	F1-Score	FPS	Inference time (ms)
**YOLOv13**	**0.858**	0.543	0.636	0.292	0.665	1428	0.7
**YOLOv13-BSAM**	0.828	**0.57**	0.648	0.299	0.675	**1667**	**0.6**
**YOlOv13-PConvHead**	0.809	0.55	0.653	0.311	0.655	**1667**	**0.6**
**YOLO-BP**	0.853	0.56	**0.665**	**0.313**	**0.676**	1250	0.8

The baseline YOLOv13 model achieves the highest precision of 0.858, with 1428 FPS but the lowest recall (0.543), mAP@0.5 (0.636) and mAP@0.5:0.95 (0.292), which suggests limited performance in detecting electrode cap tip surface defects.

The YOLOv13-BSAM model, with BSAM integrated into the backbone, achieved improvements over the baseline in terms of Recall (0.57), F1-Score (0,675), mAP@0.5 (0.648) and mAP@0.5:0.95 (0.299), and the inference speed increases to 1667 FPS. This demonstrates that BSAM enhances saliency representation of what the object is during the feature extraction stage by using fine- and coarse-grained attention, thereby improving detection performance on multi-scale and high variation defects. It aligns with results of other BRA-based models, where the accuracies are improved by enhanced feature representation through capturing both local and global dependencies [60,61]. The YOLOv13-BSAM model shows the highest Recall (0.57) indicates that BSAM is most effective in improving the true positive and reduces the false negative for detection of electrode cap tip defects.

The YOLOv13-PConvHead model, with PConv applied in the detection head, achieved higher mAP@0.5 (0.653) and mAP@0.5–0.95 (0.311) compared to the baseline and YOLOv13-BSAM models. It suggests that partial convolution effectively improves the precision of bounding box regression by improving feature sparsity and reducing noise in prediction head, which is consistent with the findings in [62,63]. YOLOv13-PConvHead maintains similar high inference speed with YOLO13-BSAM, reflecting that applying a standard convolution only to a subset of the input channels improves the detection speed without compromising the accuracy [52,53].

The proposed YOLO-BP model combines both BSAM in the backbone and PConv in the detection head, resulting in the best overall performance. It achieves the highest mAP@0.5 of 0.665, representing an improvement of 2.9% over the baseline, the highest mAP@0.5-0.95 of 0.313, an improvement of 2.1% over YOLOv13, and the highest F1-Score of 0.676, an improvement of 1.1% over the baseline. Although the precision (0.853) is slightly lower (0.5%) than the baseline, YOLO-BP maintained strong performance in Recall (0.56), and F1-Score (0.676), demonstrating effectiveness of the complementary effect of the hierarchical and selective attentions. The results indicate that integrating the BSAM hierarchical attention exclusively within the backbone is insufficient for optimal performance. Similarly, incorporating the PConv module as a selective attention solely within the detection head, where final predictions are made, also proves insufficient. However, this latter approach demonstrates improved prediction accuracy in terms of mAP compared to the exclusive inclusion of BSAM in the backbone. Previous research has highlighted that excessive use of attention in backbone position can lead to unnecessary complexity, undermining both speed and accuracy [59]. In contrast, attention in the head refines features used at the prediction stage, making it a more efficient enhancement [62,63]. Although the inference speed of YOLO-BP (1250 FPS) was lower than the baseline due to the combined computational cost of both attention modules, it still falls within the range of efficient real-time detection of defects in electrode cap tip surface in RSW. Overall, the ablation study validates the effectiveness and robustness of the proposed enhancements for improving detection capability of YOLO-BP, particularly in handling small, multi-scale and high variation defect detection tasks.

**Fig 9:**
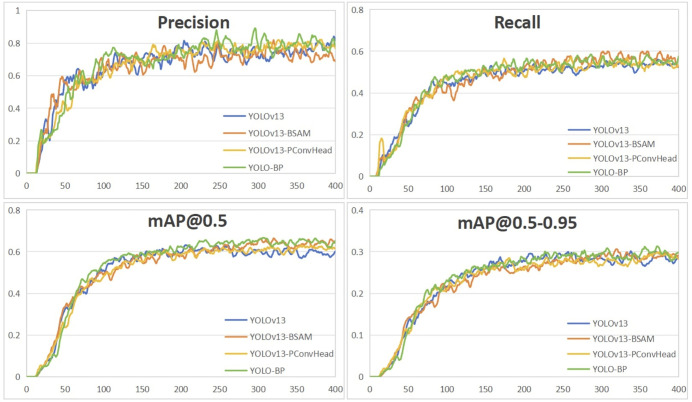
Comparison of detection results over epochs on the ECTSD Dataset.

The comparison of training process for 400 epochs on the ECTSD dataset is shown in Fig 9. As training progresses, the proposed YOLO-BP model displays a more rapid growth in Recall, mAP@0.5, and mAP@0.5–0.95 compared to the baseline YOLOv13 model and its single-module variants, clearly demonstrating the effectiveness of the proposed BSSA.

**Fig 10:**
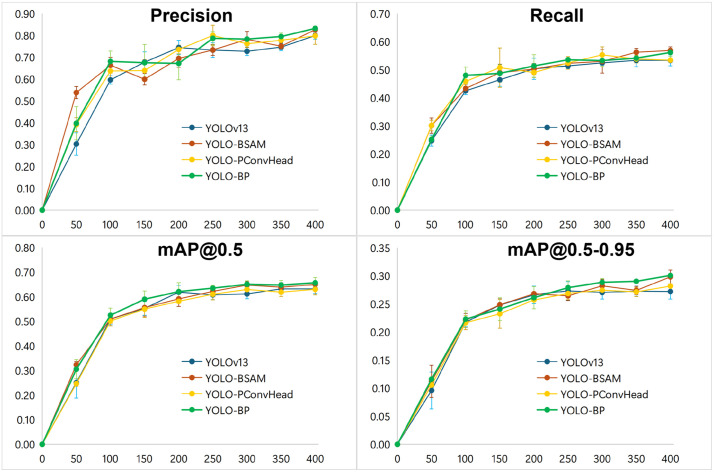
Variability comparison across independent training runs for performance metrics of models at 50-epoch intervals. Data points and error bars indicate mean and standard deviations respectively.

To evaluate the performance stability and reproducibility of the experimental results of YOLO-BP, its single-module variants and the baseline model YOLOv13, three independent training runs are conducted for each model. The convergence curves of Precision, Recall, mAP@0.5, and mAP@0.5-0.95 are shown in Fig 10, with error bars representing the standard deviation of the corresponding performance metrics across independent training runs at 50-epoch intervals. Error bars suggest greater variability during earlier epochs, with reduced variability in later epochs, indicating increased performance consistency over time for all the models. The proposed YOLO-BP demonstrates higher stochastic stability throughout the training process, specifically the tight error bars for mAP@0.5 and mAP@0.5-0.95 in the final stages of training, indicating the performance gains of YOLO-BP are highly reproducible. The result is supported by [64] that YOLOv13 exhibits stable training behavior compared to YOLOv8 through YOLOv12.

**Fig 11:**
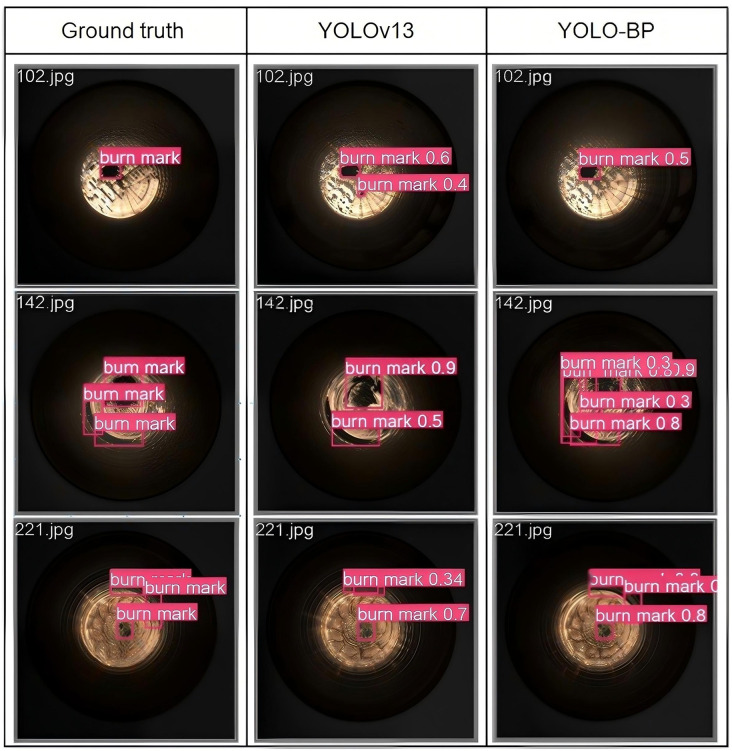
Detection result of electrode cap tip surface defect dataset.

Fig 11 presents a visualization comparison between the detection results of the proposed YOLO-BP model and the baseline YOLOv13 model on three selected images as representatives of various scales and shapes of burn marks. In the first image, a prominent oval burn mark is around the center of the electrode cap tip. However, YOLOv13 mistakenly detects a false positive object as a burn mark. The misdetection highlights the limitations of the YOLOv13 model in distinguishing subtle burn marks from other defects. In the second image, both YOLOv13 and YOLO-BP detect a random patch of burn mark around the center of the electrode cap tip, but only YOLO-BP is able to accurately detect the dark curved stripe of burn mark at the bottom left edge of the cap tip completely. This result shows the effectiveness of YOLO-BP in detecting burn marks with varying shapes and scales, demonstrating its enhanced capability to localize and classify burn marks more precisely. The third image reinforces this advantage of YOLO-BP by highlighting the detection of a light dark curved stripe of burn mark at the top right edge. The YOLOv13 model detects part of this burn mark but fails to detect the full extent of the burn mark, which results in a false negative. YOLO-BP detects the entire curved stripe of burn mark accurately, further demonstrating its advantage in reducing false negative compared to the baseline model. The reduction in false negative is crucial in practical applications, where incomplete detection of important features can lead to faulty conclusions. The visualization comparison shows that YOLO-BP model outperforms YOLOv13 by improving detection accuracy, reducing false positive and false negative, and providing a more effective solution for inspecting burn mark defects on electrode cap tips which present in diverse forms.

### Comparative study

To further validate the effectiveness of the proposed YOLO-BP, it is compared with widely recognized baseline models of YOLO, including YOLOv5 through YOLOv12. To validate the effectiveness of the proposed BSSA attention, CBAM is considered by incorporating it into YOLOv13.

The results of quantitative comparison across accuracy- and efficiency-based metrics involving YOLO-BP, YOLOv13-CBAM which incorporates attention mechanism CBAM with YOLOv13, and baseline YOLO variants (v5 through v13) are shown in Table 3. It indicates that the proposed YOLO-BP achieves a balanced optimization of both accuracy and efficiency. YOLO-BP achieves the highest F1-Score of 0.676, reflecting an improved balance between precision and recall, while also maintaining competitive mAP@0.5 (0.665) and mAP@0.5-0.95 (0.313), not at the expense of computational efficiency (FPS 1250 at 0.8 ms). While YOLOv9 yields a marginally higher mAP@0.5 (0.672), mAP@0.5-0.95 (0.324) and recall (0.57), it suffered from significant computational latency, operating at only 32 FPS. The finding is consistent with prior work [64] in comparing YOLOv8 to YOLOv13, which reports that YOLOv9 attains the highest detection accuracy but is less suitable for latency-critical applications, whereas YOLOv13 demonstrates a more balanced performance characterized by high precision and strong recall. At the opposite extreme, YOLOv12 prioritizes computational efficiency, achieving ultra-high throughput and minimal latency (FPS 2500 at 0.4 ms), but at reduced mAP (0.626) and F1-score (0.620), indicating weaker localization accuracy and class discrimination. The comparison with YOLOv13-CBAM reveals the superiority of the proposed BSSA over CBAM as attention mechanism to detect diverse multi-scale defects. YOLOv13-CBAM exhibited notably inferior performance than YOLO-BP in all metrics: lower accuracy metrics (mAP@0.5 of 0.62 and F1-Score of 0.616) and slower inference speed (1111 FPS). The sequential channel and spatial attention of CBAM could be limited by its relatively simple focus on local feature refinement, potentially inefficient in capturing features of diverse multi-scale defects. In contrast, the hierarchical attention of BSAM retains both long-range dependencies and local structural details, enhancing feature representation. Although CBAM often enhances detection performance, the integration of CBAM does not enhance performance of YOLOv13 for detection of diverse multi-scale defects. The degradation of YOLOv13-CBAM across all the performance metrics suggests that attention modules could introduce unnecessary complexity when not aligned with specific feature characteristics.

**Table 3:** Results of comparative analysis between YOLO-BP and other models.

Models	Precision	Recall	mAP@0.5	mAP@0.5-0.95	F1-Score	FPS	Inference time (ms)
**YOLOv5**	0.71	0.562	0.629	0.288	0.627	46	21.4
**YOLOv6**	0.814	0.51	0.606	0.287	0.627	1666	0.6
**YOLOv7**	0.686	0.52	0.595	0.252	0.592	238	4.2
**YOLOv8**	0.794	0.52	0.640	0.286	0.628	263	3.8
**YOLOv9**	0.811	**0.57**	**0.672**	**0.324**	0.669	32	31.2
**YOLOv10**	0.686	0.52	0.595	0.252	0.592	238	4.2
**YOLOv11**	0.807	0.53	0.642	0.303	0.640	1250	0.8
**YOLOv12**	0.767	0.52	0.626	0.291	0.620	**2500**	**0.4**
**YOLOv13**	**0.858**	0.543	0.636	0.292	0.665	1429	0.7
**YOLOv13-CBAM**	0.717	0.54	0.62	0.233	0.616	1111	0.9
**YOLO-BP**	0.853	0.56	0.665	0.313	**0.676**	1250	0.8

Overall, the superior performance of YOLO-BP suggests that its BSSA attention optimizes both detection of diverse multi-scale defects and inference efficiency. The BSSA offers advancement over baseline YOLO refinements. The findings validate YOLO-BP is a practical and novel solution for real-time detection of electrode cap tip surface defects.

## Conclusion

This paper addresses the challenges of detecting burn mark defects on electrode cap tip surface, particularly due to the large variations in target scales and shapes, as well as the limited representation of small-object features. An improved detection model, YOLO-BP, built upon YOLOv13 framework, with incorporation of a novel attention mechanism BSSA is proposed. Overall, the proposed BSSA jointly leverages hierarchical and selective attentions, enabling more effective modelling of the diverse multi-scale defect characteristics. The experiment results demonstrate that YOLO-BP outperforms the baseline YOLOv13, achieving 2.9% increase in mAP@0.5 and 2.1% increase in mAP@0.5-0.95. In addition, YOLO-BP surpasses its single-module variants, confirming the superiority of the combined attention design. Compared with YOLOv13 integrated with the CBAM attention mechanism, YOLO-BP further exhibits superior detection efficiency on the electrode cap tip surface defects, yielding gains of 4.5% in mAP@0.5 and 8% in mAP@0.5-0.95. These results validate the effectiveness of the proposed YOLO-BP for detecting diverse multi-scale defects. Future work will explore further by extending the ECTSD Dataset with broader defect categories, such as grinding marks, and enlarging its scale to improve representativeness and generalization. Moreover, more efficient and robust detection models will be investigated to achieve accurate recognition and real-time monitoring of electrode cap defects under more complex industrial scenarios.
